# A virtual reality-based intervention for surgical patients: study protocol of a randomized controlled trial

**DOI:** 10.1186/s13063-021-05196-7

**Published:** 2021-04-19

**Authors:** Raluca Diana Georgescu, Anca Dobrean, Cristina Alina Silaghi, Horatiu Silaghi

**Affiliations:** 1 The International Institute for The Advanced Studies of Psychotherapy and Applied Mental Health, Babeș-Bolyai University, Cluj-Napoca, Romania; 2 Evidence-Based Psychological Assessment and Interventions Doctoral School, Babeș-Bolyai University, Cluj-Napoca, Romania; 3 Department of Clinical Psychology and Psychotherapy, Babeş-Bolyai University, Republicii Street 37, 400015 Cluj-Napoca, Romania; 4 Department of Endocrinology, “Iuliu Hațieganu” University of Medicine and Pharmacy, Cluj-Napoca, Romania; 5 Department of Surgery, V-th Surgical Clinic, “Iuliu Hatieganu” University of Medicine and Pharmacy Cluj-Napoca, Cluj-Napoca, Romania

**Keywords:** Postsurgical pain, Virtual reality, VR-based interventions, Randomized controlled trial, RCT

## Abstract

**Background:**

Pain after surgery is normal, and treatments, including both pharmacological and psychological components, are fundamental for proper postoperative care. While several trials have investigated the analgesic effect of traditional non-pharmacological treatments, such as cognitive behavior therapies, the newer ways of delivering psychological interventions for pain after surgery are scarcely investigated. The aim of this randomized controlled trial (RCT) is to determine if delivering the psychological content through virtual reality (VR) along with the standard pharmacological treatment return better pain relief outcomes than standard care in adult patients following surgery.

**Methods:**

This is a protocol of a parallel RCT conducted in one community hospital. In order to test the efficacy of VR environments for reducing pain intensity, in the following day after surgery, adults (18 to 65 years) will be randomly assigned to either (1) standard treatment after surgery (control group) or (2) VR based intervention along with standard treatment. It is intended that a minimum of 30 patients be recruited in each group. For estimating the intensity of pain, both self-report and physiological measures will be used. Repeated measures of pain outcomes will be taken before and after the intervention. Moreover, for allowing an in-depth investigation of the effect of VR environments, the primary outcome will be complemented with measures of the adverse effects, level of immersion, and level of presence in the VR environment.

**Supplementary Information:**

The online version contains supplementary material available at 10.1186/s13063-021-05196-7.

## Background

As defined by the International Association for the Study of Pain (IASP), pain is an unpleasant sensory and an emotional experience associated with actual or potential tissue damage or described in terms of such damage [1]. Often, acute pain results from trauma or surgery and represents a warning of the brain that noxious stimuli are present or tissue is continually affected [2]. Whereas symptoms of acute pain decrease after cessation of painful stimuli, chronic pain symptoms remain beyond the recovery expected time and often continue despite treatment of initial tissue damage [1]. A report [3] based on data from National Health Interview Survey (NHIS) Sample Adult Core and the NHIS Adult Functioning and Disability Supplement pinpointed that in the year of 2012, 86.6 million US adults reported pain in some days in the last 3 months while 25.5 million reported having chronic (daily) pain. Similarly, a subsequent analysis conducted on 300 post-surgical patients [4] relived that up to 85% of people experienced acute postoperative pain, respectively pain severity was rated as moderate, severe, or extreme in 75% of those cases. Besides, acute postoperative pain was associated with prolonged hospitalization [5], alveolar ventilation, tachycardia, insomnia, and flawed wound healing [6], as well as with an increased risk of chronic post-surgery pain (CPSP) [7]. Described as the pain which persists beyond the expected recovery time after a surgical procedure [8], CPSP is reported in 10% to 70% of cases depending on the type of surgery [7, 9, 10], and several biological and psychological factors [11, 12].

Despite that in the last years pain management gained a lot of attention in both research and practice areas with enormous gains, the management of postoperative pain remains a tremendous challenge with high costs for patients and health care providers [4]. Current clinical guidelines for postoperative pain management recommend evidence-based treatments including pharmacological and psychological components [5]. The large majority of non-pharmacological components for postoperative care are interventions based on cognitive behavioral therapy (CBT) which had proved their efficacy in decreasing pain and distress in different surgical settings, such as lumbar spinal fusion [6, 7], abdominal surgery [8], cardiac surgery [9], or orthopedic surgeries [10]. These results were complemented with the findings of a recent meta-analysis [11] estimating the effectiveness of psychological interventions on postoperative pain management after orthopedic surgery, with medium effect sizes for decreasing pain intensity (Hedges’*g =* 0.31, 95% confidence interval (CI) 0.14 to 0.48) as for improving recovery (Hedges*’g =* 0.38, 95% CI 0.22 to 0.54). However, these CBT interventions were delivered into face to face format and consequently associated with several threats regarding their applicability on a large scale. Specifically, one of the biggest challenges of the face to face interventions is to synchronize the hospitalization time and patients’ availability, as some were designed to be implemented in pre-surgical settings. Likewise, other interventions need several sessions to be implemented before producing positive effects, and often patients did not have this amount of time during their hospitalization. As a consequence, most of the CBT interventions are applied only in the management of chronic pain while the treatment of acute pain remained mainly pharmacological [12]. Further, the consequences of treating pain mainly through pharmacological options are the array of side effects derived from opioids consumptions such as vomiting, nausea, respiratory depression, and physical dependence [7, 8] which are increasing the costs of pain care and place pain medicine in a peerless crisis [9].

One potential approach for increasing the applicability of CBT interventions in cases of acute pain during short hospitalization could be to develop shorter protocols, respectively to deliver the psychological content through technology, such as virtual reality (VR). The VR technology already proved its effectiveness in the case of adults in other hospital settings, such as burn units [13–15] or cancer units [16–19], and is currently under evaluation for pain relief during venipuncture in pediatric patients [20].

By using a combination of technologies (i.e., head-mounted display-HMD, vibro-tactile gloves, individualized sounds, and gesture-sensing joysticks), VR environments create immersive [21] and multi-sensorial experiences [22]. Immersion in the virtual world is believed to facilitates the shifting of attention away from the painful stimuli or from the experience of pain, to more engaging or enjoyable stimuli, developing effective distraction strategies and reshaping the pain perception [23, 24]. A recent meta-analysis [25] estimating the efficacy of VR interventions for acute pain management in clinical settings found a medium effect size of these interventions (standard mean differences (SMD) *=* − 0.49, 95% CI − 0.83 to − 0.14). Moreover, a subsequent meta-analysis [26] relying only on randomized controlled trials (RCT) conducted in hospital settings showed significant effects of VR-based interventions to reduce pain intensity during medical procedures (7 studies, Hedges’*g* = 1.09, 95% CI 0.25 to 1.92) and after the medical procedures (12 studies, Hedges’*g* = 1.08, 95% CI 0.46 to 1.70). Moreover, comparable results were founded when interventions aimed to reduce the cognitive (8 studies, Hedges’*g* = 0.82, 95% CI 0.39 to 1.26) or affective components of pain (14 studies, Hedges’*g* = 0.55, 95% CI 0.34 to 0.77). However, these promising results of VR interventions integrated into acute pain management are hampered by the fact that none of the analyses includes studies on post-surgical patients. In spite that the VR technology could be effectively exported in medical care settings [25] as a potentially cost-effective tool [27], to date, in postoperative care, only a few studies examined its efficacy. In this sense, the pain literature discusses the findings of Mosso-Vazquez and colleagues [28] which showed that 30 min of VR exposure can reduce pain intensity after cardiac surgery regardless if the pain relief was self-reported or assessed through physiological measurements (i.e., breathing rate, arterial pressure, and heart rate). Similarly, a subsequent study of Mosso-Vazquez and colleagues [29] comparing two different VR devices (i.e., HMD and mobile VR) for pain relief during ambulatory surgery revealed that both technologies decreased pain intensity, with better results for VR environments delivered through HMD. This result is consistent with other results proving that high-quality VR environments create a better distractor from pain [30] although the estimates were not in postoperative settings. Nevertheless, while these studies conducted by the Mosso-Vazquez and colleagues [28, 29] had positive and clinically significant results, highlighting the potential usefulness of VR in postoperative pain management, these studies were uncontrolled and possibly accompanied by overestimations of the VR efficacy. As far as we know, to date, there are no studies estimating the effectiveness of VR for postoperative pain following surgery under general anesthesia in a randomized clinical manner. In addition, the VR contents used in previous studies were intended only to distract patients from pain. Using environments that promote relaxation stimuli rather than distraction stimuli can be particularly useful because a relaxed state of mind was associated with a reduced demand of tissue oxygen and a reduced level of lactic acid, both being harbingers of a decreased level of pain [31]. In addition, relaxation was associated with an increased level of endorphins, lower levels of anxiety, and skeletal muscle tension. As such, the present protocol proposes a randomized controlled trial, to assess the effectiveness of relaxing VR environments for pain relief at patients in the first days after surgery.

## Objectives

The aim of this study is to assess whether exposure to relaxing VR environments is associated with decreased levels of pain after surgery after varicose veins, hernia repair, or gallbladder surgeries. Surgeries of varicose veins, hernia repair, and gallbladder were aggregated due to similarities across (1) the incidence rate [32–34], (2) levels of pain intensity after surgery [35–37], and (3) odds of acute pain to be translated into CPSP [35–37]. We hypothesize that VR-based intervention will have a better result in decreasing pain when it is used as a complementary treatment of standard care compared with the standard care alone. Additionally, we will assess the safety of VR-based intervention in patients after surgery.

## Methods and analysis

### Study design

This study is a prospective, randomized controlled trial with two parallel groups. After randomization, participants will receive either (1) standard care after surgery (control group) or (2) VR-based intervention along with standard care treatment (experimental group).

The study protocol follows the Standard Protocol Items: Recommendations for Interventional Trials (SPIRIT) [38] instructions and received the ethical approval from the Babes-Bolyai University committee as from the committee of Municipal Hospital of Cluj-Napoca and was retrospectively registered on ClinicalTrials.gov (NCT03776344).

### Study sample size

Using G*Power 3.1.9.2 [39], we had estimated a minimum of 50 participants (25 in each group) needed to detect an effect size of 0.49, with *α* = 0.05 and power = 0.80. The expected effect size of 0.49 was chosen in the light of the results found by the most recent meta-analyses [25, 26], being a conservative effect size given that in their analysis Chan and colleagues [25] found a medium effect size (SMD = − 0.49, 95% CI − 0.83 to − 0.14) and the analysis of Georgescu and colleagues [26] a large effect size was found (Hedges’*g* = 1.08, 95% CI 0.46 to 1.70) for studies with parallel design aiming to decrease pain at hospitalized patients. However, as we expected some dropouts (based on other studies using VR systems for treating anxiety [40]) and some incomplete or unusable data, especially on the physiological measure, we aim to recruit 30 participants in each group.

### Participants

All the participants were recruited from one community hospital from Romania. Starting October 2018, each patient admitted to the hospital for surgery was screened for eligibility criteria to this trial.

### Inclusion/exclusion criteria

The below criteria had to be cumulative met in order that a participant be included in the trial.

#### Inclusion criteria

- ❖ Adults aged 18–65 years, after surgery of varicose veins, hernia repair, or gallbladder surgery.
- ❖ Patient in the acute care units, 1–3 days following surgery.
- ❖ Willing and able to provide informed consent and participate in the study visit and study follow-up questionnaire.

Presence of any criteria listed below will conduct at the exclusion of the participant from the trial.

#### Exclusion criteria

- ❖ Patients with neoplastic pathologies.
- ❖ Patients with a history of motion sickness.
- ❖ Patients with severe visual impairment (i.e., not able to clearly see without glasses; patients with contact lenses will not be excluded).
- ❖ Patients with severe/profound cognitive impairments measured with Six-item Cognitive Impairment Test (6CIT).
- ❖ Use of strong opioids (i.e., morphine)
- ❖ Other reasons for exclusion (Non-Romanian speaking patients, patients with severe psychological problems, etc.)

Some of the exclusion criteria were dependent on the VR device or were designed as a precautionary measure for not harming participants after using VR technology. Particularly, we excluded the participants with profound visual impairments (i.e., those which cannot clearly saw without glasses or without contact lenses) due to the incompatibility with the VR device which was not equipped with the eyes glasses spencer. Similarly, patients with a history of motion sickness were excluded for preventing any severe dizziness or injury.

### Randomization and blinding

Randomization was conducted within the type of surgery using a random number generator, with an equal number of participants in the control and experimental group. An independent researcher had conducted the randomization sequence and the allocation sequence was stored on a secured computer until the participants were assigned to one of the interventions. The medical personnel and trial assessors were blind to the group allocation, but the study participants and research assistant were aware of the allocations. Precaution strategies to prevent the blind from breaking up included a limited number of persons interacting with the patients (i.e. only the research assistant) and a reminder to the patients not to talk about research allocations and study procedure. In addition, the treatment room was a different room of the patient hospital room. In the treatment room patients from the experimental group was exposed to the VR content and psychological respectively physiological measured. For the patients from the control group, only psychological and physiological measures were taken without exposing them to the VR content. After this, each participant was conducted to their hospital room. In the case that a participant explicitly requests to end the study procedure for any reasons, the procedure was stopped and counted as a dropout. Those patients who ask to end the procedure earlier were asked to respond at a short interview to quantify the reasons.

### Recruitment procedure and interventions

**Fig. 1:**
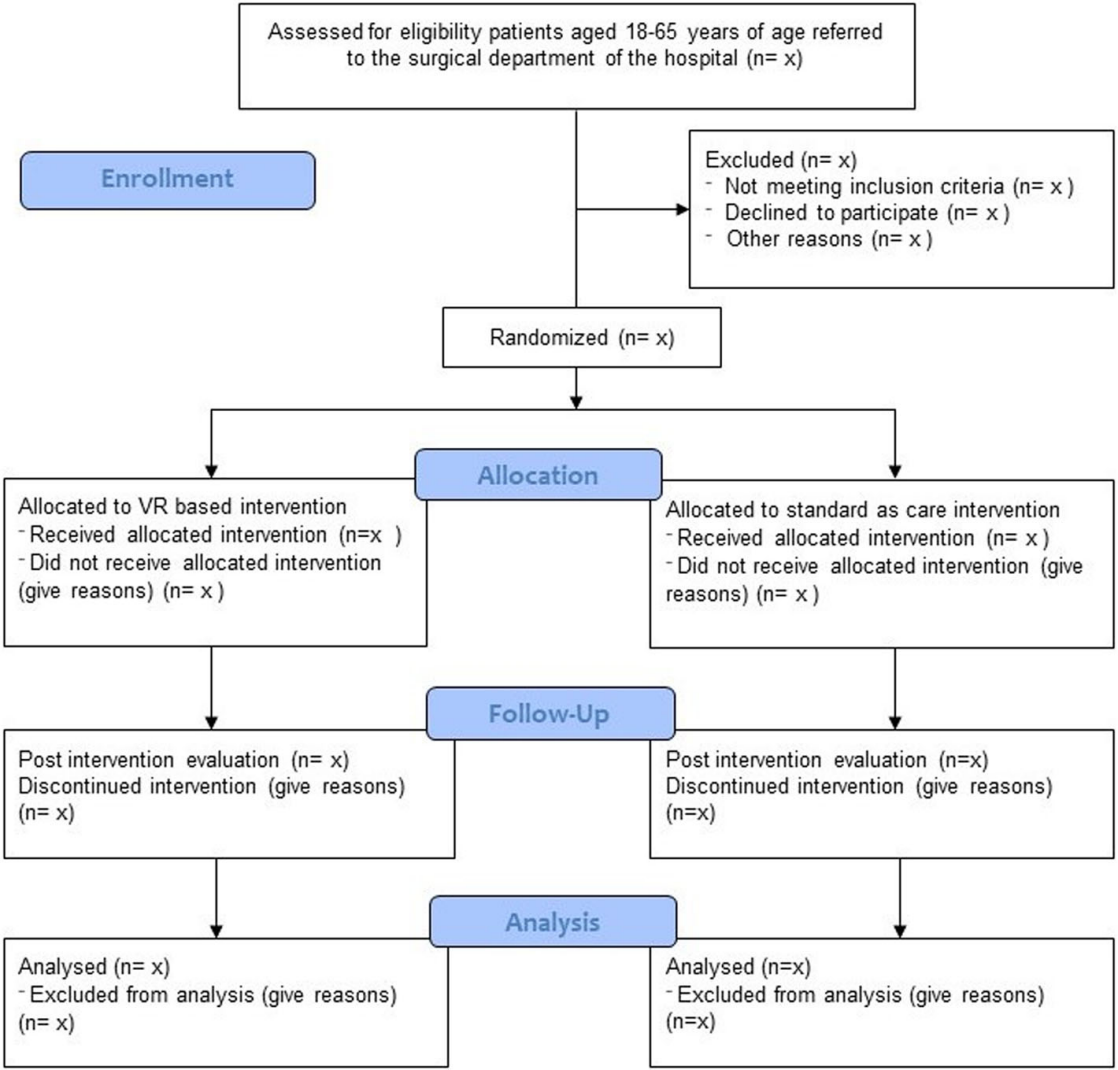
Flowchart of the study based on the Consolidated Standards for Reporting of Trials

The recruitment process and data collection are presented in Fig. 1. No specific strategy for improving adherence was planned. The day following the surgical procedure, all patients from the acute care unit who meet the primary criteria for inclusion (i.e., age, type of surgery, type of opioids used, free of visual impairments, able to fluently speak in Romanian and without recorded psychological problems) were invited to participate. Information regarding duration, procedure, implications, and conditions for withdrawing were presented and explained. To the interested patients, the research assistant presented the informed consent asking to read and sign as a final statement regarding their decision to enroll in the study. Subsequently, those who have signed the informed consent were invited to the treatment room where they have completed the Six-item Cognitive Impairment Test for assessing the eligibility regarding executive functions. Finally, those patients with intact executive functions were randomly allocated to one of the two groups:

#### Treatment group: VR-based intervention

Patients allocated to the VR-based intervention followed the standard protocol after surgery as prescribed by the current medical personnel and exposed for 15 min to an interactive virtual environment (i.e., Nature Treks© VR). This application is a commercially available app from the Oculus store (available at https://www.oculus.com/experiences/go/1723271804396968/), promoting relaxation through fifteen highly immersive environments. Each environment recreates a different natural scene (e.g., a tropical beach, savannas at sunset, snowy forests) which can be explored by the patients through a controller. Concomitant with the activities (e.g., walking on the beach, climbing the mountains) environmental effects are changing smoothly to create a vivid experience. Additionally, in some environments (i.e., deep blue and black beginning), patients can freely explore the scenes in 360 degrees for enhancing the feeling of presence and immersion.

As previous studies testing the efficacy of the analgesic effect of VR [30] showed that better immersion is associated with lower scores for pain intensity, the device used is an Oculus Rift® (available at - https://www.oculus.com/). This device is the premium device from Oculus, equipped with a highly immersive headset, one controller, and integrated headphones.

Five minutes before and during the VR exposures, the fluctuations of skin conductance (SC) were measured for all patients. Before and after the intervention, pain intensity, relaxation, and VR adverse effects were assessed. Additionally, the catastrophizing level, anxiety, and depression related to health and presence in the VR environment were quantified.

#### Control group: standard of care intervention

Patients allocated to the standard of care group followed the treatment after surgery as prescribed by current medical personnel. In addition, they followed the same protocol as the patients in the intervention group regarding psychological and physiological measures. Specifically, they were conducted in the treatment room and asked to indicate the non-dominant hand for setting up the equipment to SC measures. After that, the SC was measured continuously for 20 min without exposure to the VR environments. At the end, they completed de psychological measures and were conducted in their hospital room.

### Data collection procedure

**Fig. 2:**
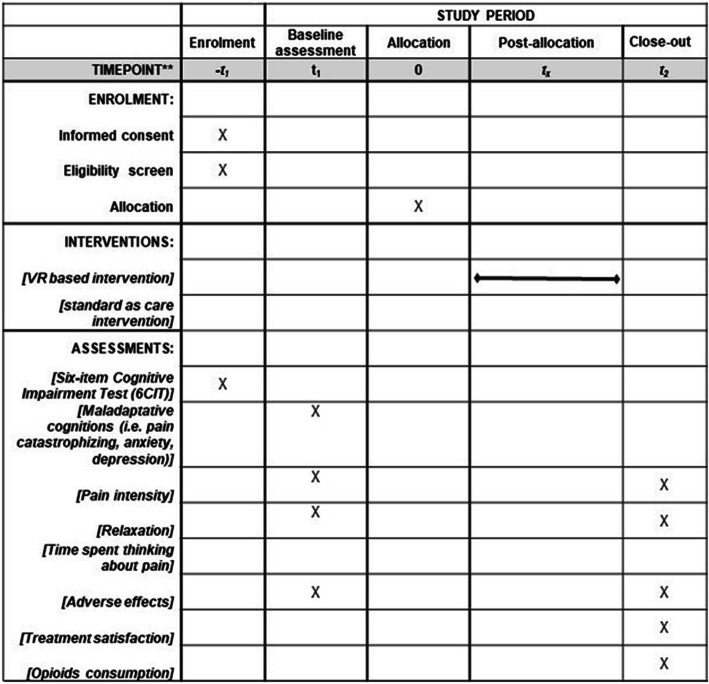
Overview of the enrollment, intervention, and assessment measures

Figure 2 offers an overview of the process of the data collection and measures. There is no anticipated harm and compensation for trial participation. This trial does not involve collecting biological specimens for storage. All psychological and physiological measures were collected by a previously trained researcher which had weekly meetings with RG and HS to discuss the potential difficulties and assess the adherence to the study protocol. The level of pain intensity and relaxation was collected before and after the exposure to VR environments in the experimental group, respectively before and after physiological measures in the control group. The SC was measured during the exposure to VR content in the experimental group and for a period of 15 min in the control group. For ensuring an accurate baseline for the physiological measure and for controlling the individual differences in SC, the signal of SC for each participant was taken before the study procedure start for 5 min. During this time, participants had no other instructions, and the communication was maintained at the minimum level. The amount of analgesic consumption for each participant will be extracted from the medical records. Excepting for the fluctuations of skin conductance, all measures were collected through an online platform.

### Outcomes

The present study assesses the efficacy of a Nature Track© VR to decrease pain intensity in surgical patients. The primary outcome is pain intensity measured right before the exposure to the VR content and after. The secondary outcomes measure the effects of the application on relaxation and the amount of time spent by each patient thinking about pain. Also, the primary outcome was complemented with measures of the adverse effects, level of immersion, and level of presence in the VR.

## Measures

### Primary measures

#### Pain

The pain intensity was measured using the Numerical Rating Scale (NRS) [41] by asking participants to report their intensity before and after the intervention on a scale from 0 (“no pain”) to 10 (“extremely painful”). To help patients to discriminate between different pain levels, they were asked to report the mean level of pain intensity in the last 24 h, the peak of intensity in the same period, and the intensity right before the intervention. We chose NRS for this study rather than other measures more extensively used, such as visual analog scales (VAS), due to the consensus of the better psychometrics properties [42–45]. The value of pain right before the intervention will be used in the analysis of VR effectiveness.

Concomitantly, as a physiological indicator of postoperative pain were measured the fluctuations of the SC [46–48]. The SC were measured using the BIOPAC MP150 system (Biopac Systems, CA,USA). The two finger electrodes were attached to the first phalange of the index and medius fingers from the non-dominant hand with Velcro straps. The electrodes were connected to the computer using the USB connection input. To ensure good contact with the skin, isotonic gel was added to the electrodes prior to attaching to the fingers. At the beginning, participants had few minutes to relax, get ready and comfortable with the experimental environment. After that, patients were instructed not to move for the duration of the recording.

### Secondary measures

#### Relaxation

As the level of relaxation could affect the perception of the pain intensity, the state of relaxation was measured using the NRS from 0 (“extremely stressed” to 10 (“relaxed”) before and after the intervention. We chose to measure by using NRS rather than other scales designed to measure relaxation due to his factual effect and similarities with the pain intensity measures.

#### Time spent thinking about pain

Another factor which can contribute to an increased perception of pain intensity is the amount of thinking time spent by a patient at pain [49]. Consequently, we asked patients to report after the interventions, on NRS from 0 (“not at all”) to 10 (“all the time”), the amount of time they spent thinking about their pain during the exposure to the VR content in the experimental group and during the measuring of SC conductance in the control group.

#### Adverse effects

Potential adverse effects were evaluated using the Simulator Sickness Questionnaire (SSQ) [50]. As some of the unintended effects of VR could also be effects of the opioid’s consumptions (e.g., headaches, nausea), we have asked participants to complete the scale twice, before and after the exposure of VR. The SSQ was previously validated and proved robust psychometric properties [50, 51] being the most widely measure of cyber-sickness. The patients were instructed to answer on a 4-point Likert scale, corresponding to not at all, slight, moderate, and strong sensations regarding the occurrence of possible side effects such as general discomfort, fatigue, headache, and dizziness.

#### Treatment satisfaction and presence

The presence in VR was assessed using Igroup presence questionnaire (IPQ). This is a 14 items questionnaire assessing different aspects of presence and immersion into VR world through items such as: “In the computer generated world I had a sense of being there” or “How aware were you of the real world surrounding while navigating in the virtual world? (i.e. sounds, room temperature, other people, etc.)?”. In the end, once the IPQ completed, the patients will be instructed to answer an additional question (i.e., are you willing to use VR systems in the future?) with dichotomous response developed by the authors for assessing the willingness for further sessions with VR system.

#### Opioids used

The amount of analgesic used will be extracted from the medical records. The usage of opioids will be coded as present and absent. The mean drug metabolism time will be calculated in order to determine if an opioid agent is active, coding 1 if the opioid agent is active and 0 if is out of his action range.

#### Pain catastrophizing

Level of catastrophizing was measured through the Pain Catastrophizing Scale (PCS) [52]. This scale is a self-report measure with 13 items structured in three subscales, namely rumination, magnification, and helplessness, and proved good psychometrics properties. Patients were instructed to answer on a scale from 0 (“not at all”) to 4 (“all the time”).

#### Assessment of mood

Anxiety and depression levels were assessed through the Hospital Anxiety and Depression Scale (HADS) [53]. This scale is a self-report measure with 14 items, with half of the items measuring anxiety symptoms (e.g., items targeting tension, panic attacks) and the other half measuring depression symptoms (e.g., items targeting anhedonia or inability to enjoy things or experiences). Responses are recorded on a scale from 0 to 3, and each item has a different response in accordance with the item content.

#### Cognitive abilities

Cognitive abilities were measured through the Six-item Cognitive Impairment Test (6CIT) [54], a screening tool for measuring the global cognitive status. The items of the 6CIT cover six questions: one assessing the memory (remembering a 5-item name and address), two items including calculation (reciting numbers backward from 20 to 1 and months of the year backward), and three items assessing orientation (year, month, and time of day). The cutoff of seven from the total score was used for excluding patients with low cognitive abilities [55].

### Statistical analysis

#### Preliminary analyses and preprocessing data of skin conductance

Demographic characteristics and psychological measures will be explored for missing data, and distribution abnormalities. Means and standard deviations will be used to characterize the sample. Preprocessing of skin conductance measure will be performed using AcqKnowledge 4.1 first through the visual inspection of the raw signal, and then applying the smoothing function with a smoothing factor of three samples. This function has the same effect as the low pass filter by replacing the high-frequency signal with the mean values across three milliseconds to subtract the artifacts, without changing the wave form.

#### Main analyses

Behavioral data will be analyzed using STATA 16.1 in accordance with the intent to treat principal [56]. Descriptive statistics (i.e., age, gender, assessment of mood, pain catastrophizing, opioid consumption, level of pain intensity, level of relaxation) for the baseline values will be presented within each randomized group. These will include counts and percentages for binary variables and mean and standard deviations, or medians with lower and upper quartiles, for continuous variables, along with minimum and maximum values and missing values. There will be no tests of statistical significance or confidence intervals for differences between randomized groups on any baseline variable. The effectiveness of the intervention on pain intensity (primary outcome) will be assessed by employing random coefficient analysis. We will include the time of assessment, group assignment (VR intervention and standard of care), and the interaction between time of assessment and group in the multi-level regression model. Similarly, for the secondary outcome’s measures (relaxation, time spent by patients thinking about pain, and treatment satisfaction and presence) random coefficient analysis will be performed. Although the intervention is minimally invasive, the adverse effects of the VR intervention will be measured with SSQ scale and pre-post differences will be assessed by *t*-test statistic or the non-parametric equivalent. Physiological data will be processed using the AcqKnowledge 4.1 software, and for each patient, a difference score in the area under the curve between the last 5 min of measurement and baseline level of SC will be extracted. Pearson correlation will be employed to examine the relationship between the level of SC and pain intensity (as a difference score between pre-post intervention). A *P* value will be used to estimate statistical significance for all analyses.

### Interim analyses

As virtual reality technology is not known to be associated with severe adverse events and because the present intervention is designed for one session, 15 min length, there has no planned interim analyses or stopping rules.

### Oversight and monitoring

From the study group, RG and HS were responsible for the supervision of data acquisition. In this vein, they had weekly meetings with the research assistant responsible for data collection to monitor the entire process. A Data Monitoring Committee was not employed as the study implementation was designed in one site and the intervention has a low risk for adverse effects. Any change from the protocol will be reported to the ethical committee of the Babeș-Bolyai University and will be updated in the ClinicalTrials.gov (NCT03776344).

## Discussion

Treating pain after surgical interventions is an overly complex and challenging process (61,62), and the integration of non-pharmacological approaches has been one of the priorities of healthcare reform in the last years [5]. Additional interventions along the standard pharmacological treatments are strongly recommended by the guidelines for postoperative pain management [5]. Limited medical personnel resources and short periods of hospitalization are widely known problems of the postoperative management, highlighting the need for additional interventions that are requiring very little set-up time to co-occur with the standard pharmacological treatment. Addressing these problems, VR interventions can be used as a non-pharmacological tool for reducing pain after surgery. The present protocol offers a detailed description of a randomized and controlled clinical trial to determine the effectiveness of VR environments in reducing pain intensity in surgical patients.

Should the hypotheses suggested in this study be proven, this would add empirical evidence about the benefits and feasibility of VR for the pain treatment in surgical patients. Our expectation is to obtain greater pain relief compared with studies employing non-technological interventions. Moreover, we expect that the results of the present study to be in line with the ones conducted by Mosso-Vazquez and colleagues [32] as well with studies that use VR for non-surgical patients [29, 30]. In addition, we expect to find minimal side effects in those patients exposed to VR and the level of willingness to use VR sessions in the future treatment of pain to be high.

However, the present protocol is exposed to several threats. First, by recruiting participants from o single hospital is possible to affect the external validity of the study. Will be unknown if the characteristics of our participants will be representative for a broader population of surgical pain suffers. Moreover, our results cannot be extrapolated to other VR environments, being strictly linked to Nature TreksVR®. Second, the present protocol uses self-reported measures to asses pain intensity, level of relaxation, mood, pain catastrophizing and an open-label design, thus is possible that some of the reports be affected by the knowledge of the group assignment. Nonetheless, the trial assessors and researchers conducting data analysis will be blinded in order to improve the internal validity of the study and maintain a low detection bias (63). Not lastly, our results will be subtracted in the absence of other non-pharmacological treatment, thus, in the case of hypothesis confirmation we can conclude that the Nature TreksVR® produce significant reductions in pain intensity but we cannot know the effects when other non-pharmacological interventions are applied or when VR-based interventions are integrated in more comprehensive treatments of pain intensity.

Concluding, although several threats exist, this study is to our knowledge, the first to test the efficacy of a modern, market-ready VR application for the treatment of pain after surgery, and his results will assist in the development of a new method for the delivery of evidence-based treatments.

### Data storage

During data collection, all the data will be safely stored at the Municipal Hospital. After the implementation phase is completed, all the data will be transferred in a secured PC at the International Institute of Psychotherapy, accessible only by the principal investigator (RG) and the corresponding author (AD).

### Dissemination

Following the recommendations of the Consolidated Standards of Reporting Trials (CONSORT) [57] statement for reporting RCTs, results will be submitted to a peer-reviewed journal and presented as oral or poster presentation at international scientific conferences. Additionally, we will make available all the results to patients if required. After the results are published in a peer-review journal, all the materials used will be made public at request. The datasets corresponding to the current study are available from the corresponding author on reasonable request.

## Trial status

The trial started patient recruitment in October 2018 and completed in September 2019. At the date when the first draft of the present protocol was submitted (March 2019) the status of data collection was “on going.” Currently, the data for opioid consumption were not extracted from the patient medical records, and data analysis was not performed. A trial paper with the results should be submitted for publication around the beginning of 2021. The present version of the protocol is 1.5 (March 2021).

## Supplementary Information

**Additional file 1.** Informed consent.

## Data Availability

Not applicable

## Abbreviations

RCT: Randomized controlled trial
VR: Virtual reality
IASP: International Association for the Study of Pain
CPSP: Chronic post-surgery pain
CBT: Cognitive behavioral therapy
HMD: Head-mounted display
SPIRIT: Standard Protocol Items: Recommendations for Interventional Trials
SC: Skin conductance
NRS: Numerical Rating Scale
VAS: Visual analog scales
SSQ: Simulator Sickness Questionnaire
SEQ: Suitability Evaluation Questionnaire
PCS: Pain Catastrophizing Scale
HADS: Hospital Anxiety and Depression Scale
6CIT: Six-item Cognitive Impairment Test
