# Supplementary material for: A virtual reality-based intervention for surgical patients: study protocol of a randomized controlled trial
Source: Trials. 2021 Apr 19;22:289. doi: 10.1186/s13063-021-05196-7 (PMC8056576; doi:10.1186/s13063-021-05196-7)
Supplement: Supplementary file 1 — Additional file 1. Informed consent. [file 13063_2021_5196_MOESM1_ESM.docx]

**Informed consent**

First, thank you for your interest! Before the study starts, it is important that you are informed about the procedure. Therefore, we would like you to read this information letter carefully.

**Goal of the study**

The goal of this study is to investigate the efficacy of different non-pharmacological approaches on the pain management.

**Procedure**

You will be asking about your background, your health and beliefs regarding pain. During the procedure your level of skin conductance will be measured and you can be exposed to a content delivered through virtual reality technology.

**Voluntary participation**

There are no consequences if you decide now not to participate in this study. During the procedure, you are free to stop participating at any moment without giving a reason for doing so.

**Your privacy is guaranteed**

Your personal information (about who you are) remains confidential and will not be shared without your explicit consent. Your research data will be analyzed by the researchers that collected the information. Research data published in scientific journals will be anonymous and cannot be traced back to you as an individual. Completely anonymized data can be made publicly accessible.

**Compensation**

There are no compensations for participating at this study.

**Further information**

Should you have questions about this study at any given moment, please contact the responsible researcher; R. Georgescu ([ralucageorgescu@psychology.ro](mailto:ralucageorgescu@psychology.ro)) . Formal complaints about this study can be addressed to the Ethics Review Board from the Babes-Bolyai University or Municipal Hospital of Cluj-Napoca.

By signing this informed consent, you acknowledge that:

- You are 18 or older;

- You have read and understand the above information letter;

- You agree to participate in this study and agree with the use of the data that are collected;

- You reserve the right to withdraw your participation from the study at any moment without providing any reason.

- I consent, begin the study
- I do not consent, I do not wish to participate

Date Signature

______________________ ___________________
